# Crystal structure of vaccinia virus uracil-DNA glycosylase reveals dimeric assembly

**DOI:** 10.1186/1472-6807-7-45

**Published:** 2007-07-02

**Authors:** Norbert Schormann, Alexei Grigorian, Alexandra Samal, Raman Krishnan, Lawrence DeLucas, Debasish Chattopadhyay

**Affiliations:** 1 Center for Biophysical Sciences & Engineering, University of Alabama at Birmingham, Birmingham, AL 35294, USA; 2 BioCryst Pharmaceuticals, Birmingham, AL 35244, USA; 3 Department of Medicine, University of Alabama at Birmingham, Birmingham, AL 35294, USA

## Abstract

**Background:**

Uracil-DNA glycosylases (UDGs) catalyze excision of uracil from DNA. Vaccinia virus, which is the prototype of poxviruses, encodes a UDG (vvUDG) that is significantly different from the UDGs of other organisms in primary, secondary and tertiary structure and characteristic motifs. It adopted a novel catalysis-independent role in DNA replication that involves interaction with a viral protein, A20, to form the processivity factor. UDG:A20 association is essential for assembling of the processive DNA polymerase complex. The structure of the protein must have provisions for such interactions with A20. This paper provides the first glimpse into the structure of a poxvirus UDG.

**Results:**

Results of dynamic light scattering experiments and native size exclusion chromatography showed that vvUDG is a dimer in solution. The dimeric assembly is also maintained in two crystal forms. The core of vvUDG is reasonably well conserved but the structure contains one additional β-sheet at each terminus. A glycerol molecule is found in the active site of the enzyme in both crystal forms. Interaction of this glycerol molecule with the protein possibly mimics the enzyme-substrate (uracil) interactions.

**Conclusion:**

The crystal structures reveal several distinctive features of vvUDG. The new structural features may have evolved for adopting novel functions in the replication machinery of poxviruses. The mode of interaction between the subunits in the dimers suggests a possible model for binding to its partner and the nature of the processivity factor in the polymerase complex.

## Background

Poxviruses are unique among DNA viruses in that their entire life cycle, including DNA replication, occurs exclusively within the cytoplasm of the host cell. Therefore, the virus does not depend on cellular nuclear functions, and relies largely on its own gene products for DNA replication, transcription and virion assembly. Proteins required for DNA replication of poxvirus are thereby expressed early in infection.

Vaccinia virus, the best characterized member of the *Orthopoxvirus *family, is used as the smallpox vaccine. Its genome of ~200 kb encodes more than 200 proteins that are highly conserved among poxviruses. The uracil-DNA glycosylase (UDG), encoded by the D4 open-reading frame (ORF), is essential for viral replication. Uracil residues are introduced into DNA either through misincorporation of dUTP by DNA polymerase or through deamination of cytosine. In general, UDGs catalyze the first step in the base excision repair pathway and remove uracil residues from DNA by cleaving the glycosidic bond, resulting in an apyrimidinic (AP) site. However, in poxvirus UDG activity is rapidly induced following infection, suggesting that the enzyme is required prior to and during DNA synthesis [1]. Two observations have indicated the involvement of vvUDG in DNA replication. The virus cannot replicate in the absence of UDG [2], and two temperature-sensitive (*ts*) mutations conferring defective DNA replication map to the D4 ORF [3]. The first *ts *mutant, D*ts*30 (*ts*4149), containing a G179R substitution was partially impaired both in virus production and DNA replication at the permissive temperature (31.5°C) while it displayed a strong DNA^- ^phenotype at the non-permissive temperature (39.7°C) [2]. The second *ts *mutant, D*ts*27 (*ts*3578), containing a L110F substitution showed normal levels of DNA synthesis and virus production at 31.5°C but DNA synthesis was essentially blocked at 39.7°C [2]. Additional support for the involvement of vvUDG in viral replication comes from the discovery that vvUDG interacts with another viral protein, A20 and forms the processivity factor [2]. The UDG:A20 complex (stoichiometry 1:1) binds to E9 (the catalytic subunit of DNA polymerase) to assemble the processive DNA polymerase holoenzyme (stoichiometry of binding 1:1:1). The protein-protein interaction between UDG and A20 is essential for viral replication. However, this interaction does not depend on the glycosylase activity or the presence of the catalytic residues in UDG [4]. The interaction site on A20 has been mapped to its N-terminal 50 residues [5] but the A20 binding site on UDG is not known.

The vvUDG enzyme is highly specific for uracil and preferentially excises uracil when present in single stranded DNA (ssDNA). Although viral UDG has a stronger affinity for ssDNA (K_M _= 0.5 μM) than the human enzyme (K_M _= 2.9 μM), the excision efficiency of the human enzyme was several orders of magnitude higher [1]. In the absence of MgCl_2 _vaccinia virus and human nuclear UDG have comparable activity, but vvUDG is strongly inhibited in the presence of MgCl_2_, while the human nuclear UDG shows markedly enhanced activity. In addition, while the human enzyme is strongly inhibited by the uracil-DNA glycosylase inhibitor protein (Ugi) from *Bacillus subtilis *bacteriophages PBS1 and PBS2, vvUDG shows no inhibition. Overall the enzymatic properties of vvUDG differ from the human enzymes suggesting a different mechanism of action. Moreover, poxvirus UDGs exhibit low sequence identity to other UDGs. Therefore, vvUDG may offer a potential target for specific inhibitors.

Here, we describe the crystal structure of vvUDG in two different crystal forms, and provide a comparison with the most studied known UDG structures (human and *E. coli*). These structures provide the first glimpse of a poxvirus UDG and show unique features that distinguish the enzyme from all other members of the UDG protein family.

## Results and discussion

vvUDG plays an essential role in viral replication as a component of the DNA polymerase processivity factor. The enzyme diverged significantly from UDGs of other species in its primary, secondary and tertiary structure, and through modifications of otherwise conserved active site motifs.

vvUDG is a single-domain protein with 218 amino acids. Results of size exclusion chromatography and dynamic light scattering showed that in solution recombinant vvUDG exists as a dimer of an estimated molecular weight of 57 kDa.

UDGs of various members within the poxvirus family show a high degree of sequence homology. UDGs from the variola (smallpox) virus and vaccinia virus differ only in 3 positions. Among all poxviruses, fowlpox UDG has the lowest sequence identity (71%) with vvUDG. On the other hand, sequence identity to UDGs from organisms outside the poxvirus family is only about 20%.

### Structure determination and quality

We have determined the crystal structure of recombinant vvUDG by SIRAS phasing in trigonal space group P3_2_21. The structure was refined to an R value of 24.1% (R_free _of 29.9%) at 2.4 Å resolution (see Tables 1 and 2). The recombinant protein used for crystallization contained an N-terminal His-tag. The final model consists of two subunits A and B with a total number of 440 protein residues (subunit A: residues -1 to 171, 174 to 186 and 189 to 218; B: -8 to 172, 174 to 186 and 189 to 218), 1 chloride and 1 sulfate ion, 146 water, 4 glycerol (GOL) and 3 imidazole (IMD) molecules. Parts of the N-terminal His-tag are visible in both subunits, A (2 residues: -1 to 0) and B (9 residues: -8 to 0). Several residues in two loop regions could not be fitted into the electron density in each subunit (A: 172,173, 187,188; B: 173, 187,188) presumably due to disorder. In addition, some residues have truncated side chain density in both subunits (A: -1, 0, 170, 171 and 195; B: -8, -6, 0, 185 and 195). 3 residues in subunit B (S7, N17 and Q203) show alternate conformations. The Ramachandran plot shows 96.3% of all protein residues in the allowed regions, with 12 residues (3.2%) in generously allowed and 2 residues (0.5%) in disallowed regions. Electron density for several of the residues in generously allowed regions (A9, Y11, F79 and N206 in both subunits) and the two residues in the disallowed region (R167 and A168 in subunit B) is good. Of these residues A9 and Y11 are part of a β-hairpin turn connecting strands 1 and 2. N206 is in the turn following helix 9 (residues 189–205) in the C-terminal part of the protein. Refinement statistics are shown in Table 2.

**Table 1:** Crystal data and data collection statistics for vvUDG.

	**Native I**	**Native II**	**Derivative (Uranyl)**
Space Group	P3_2_21	P2_1_2_1_2_1_	P3_2_21
Unit Cell Dimensions	a = 85.20 Å b = 85.20 Å c = 139.72 Å α = β = 90°, γ = 120°	a = 117.77 Å b = 134.06 Å c = 139.10 Å α = β = γ = 90°	a = 85.15 Å b = 85.15 Å c = 139.53 Å α = β = 90°, γ = 120°
Resolution Range	19.96–2.40 (2.48–2.40)	20.00–2.30 (2.38–2.30)	20.00–2.80 (2.97–2.80)
Total No. of Reflections	62227	733463	75084*
Unique Reflections	23203	98092	27719*
Average Redundancy	2.7 (2.7)	7.5 (7.5)	2.8 (2.8)
Completeness [%]	98.6 (99.5)	100.0 (100.0)	99.1 (99.8)
Reduced Chi Squared	0.99 (1.10)	N/A	N/A
R_merge _[%]	6.1 (29.9)	6.0 (25.3)	9.1 (34.2)
Mean I/σ(I)	11.0 (3.4)	10.4 (2.1)	11.3 (2.6)

The numbers in parentheses are for the highest-resolution shell.

* Friedel Pairs

**Table 2:** Refinement statistics for vvUDG.

**PDB ID**	**Trigonal (Native I) **2OWQ	**Orthorhombic (Native II) **2OWR
Resolution [Å]	19.27–2.40 (2.46–2.40)	20.00–2.30 (2.36–2.30)
No. of Reflections	21956 (1699)	93107 (7120)
Completeness [%]	98.4 (99.7)	99.9 (99.9)
R_all _[%]	24.4	25.5
R_work _[%]	24.1 (29.5)	25.3 (28.3)
R_free _[%]	29.9 (35.5)	30.2 (38.0)
No. of Atoms		
Overall	3725	14550
Protein	3534	13838
Ligands IMD, GOL, EPE	15, 24, NA	NA, 108, 30
Ions Cl^-^, SO_4_^2-^	1, 5	1, NA
Water	146	573
Wilson B-factor [Å ^2^]	35.0	26.0
Average B-factors [Å ^2^]		
Overall	27.5	20.9
Protein	27.6	20.6
Ligands IMD, GOL, EPE	37.0, 33.0, NA	NA, 30.6, 62.7
Ions Cl^-^, SO_4_^2-^	43.7, 55.5	30.7, NA
Water	24.0	23.8
R.m.s. Deviations		
Bonds [Å]	0.01	0.01
Angles [°]	1.13	1.33
Coordinate error ESU (max. likelihood)	0.26	0.21
Correlation Coefficient		
F_O_F_C_	0.92	0.90
F_O_F_C _free	0.88	0.86

The numbers in parentheses are for the highest-resolution shell.

We also crystallized vvUDG (without His-tag) in orthorhombic space group P2_1_2_1_2_1_. This structure was determined by molecular replacement using the refined model of the trigonal crystal form, and was refined to an R value of 25.3% (R_free _of 30.2%) at 2.3 Å resolution (Tables 1 and 2). The final model consists of 8 subunits A through H with a total number of 1708 residues (subunits A: 214 residues; B: 212; C: 215; D: 215; E: 216; F: 214; G: 213; H: 209), 1 chloride ion, 573 water, 2 Hepes (EPE) and 18 glycerol (GOL) molecules. Several residues in the same two loop regions could not be fitted into the electron density in each subunit (A: 185–188; B: 168,169, 186–189; C: 187–189; D: 193–195; E: 185,186; F: 164, 165, 186,187; G: 188–192; H: 171–173, 185–190) and are missing from the final model. The Ramachandran plot shows 96.4% of all protein residues in allowed regions, while 2.6% of the residues are found in generously allowed and 1.2% (18 residues) in disallowed regions. Here, the same residues (A9, Y11, F79 and N206) with good electron density as mentioned for the trigonal crystal form are also found in generously allowed and disallowed regions. Refinement statistics are shown in Table 2. The average *rms *deviation for all Cα atoms between subunits of the two different crystal forms is ~0.5 Å.

### Overall polypeptide fold

**Figure 1:**
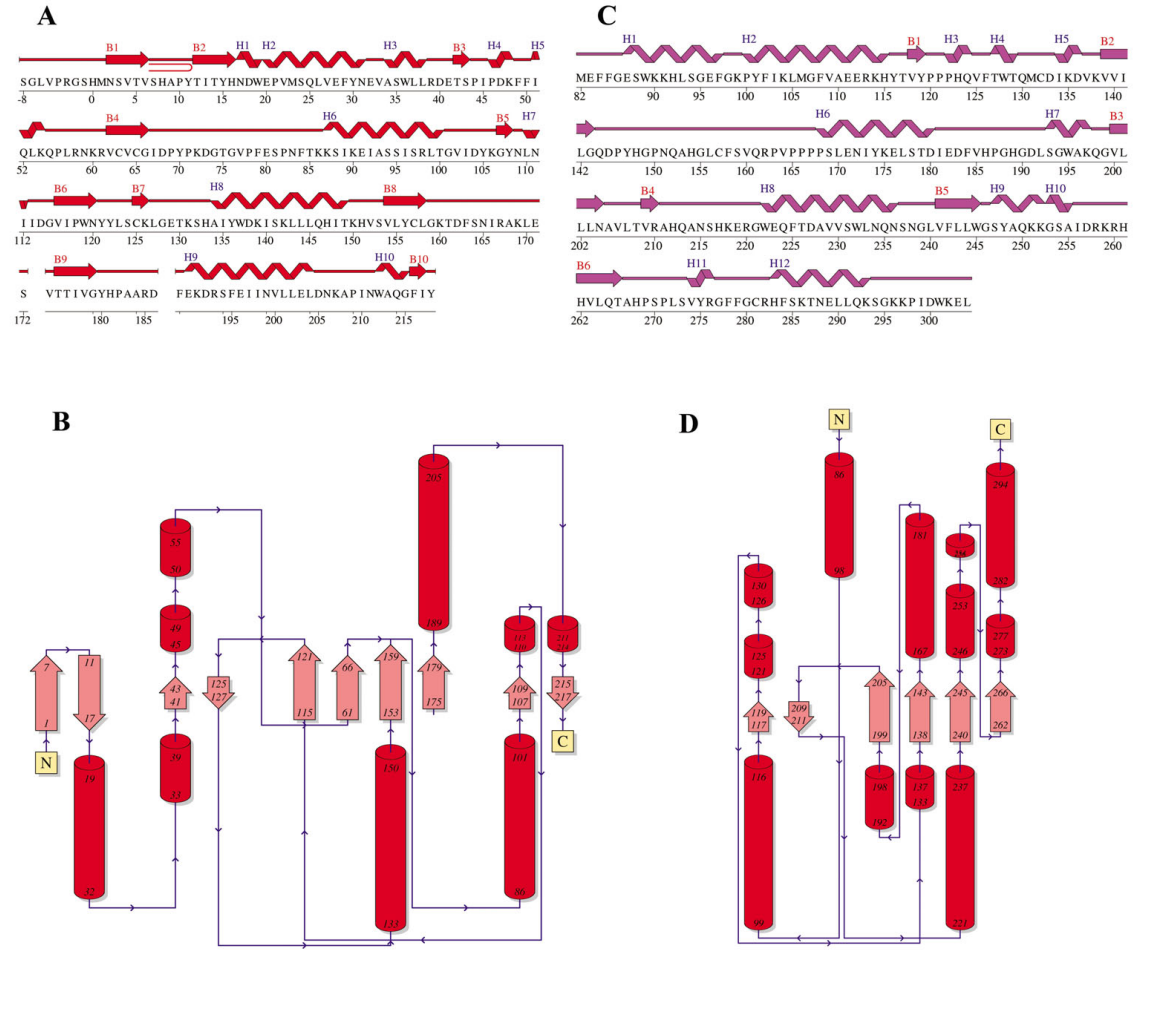
**Protein sequence with secondary structure assignments and topology diagram for vvUDG and comparison with human UDG**. (**A**) Wiring plot for vvUDG. The figure shows the protein sequence overlaid with assigned secondary structure elements for the vvUDG monomer. The β-strands are labeled B1 through B10 and α helices are labeled H1 through H10. A β-hairpin turn between the two N-terminal β-strands B1 and B2 is also shown. The portions of the missing loop regions are indicated by spaces. Several residues of the N-terminal His-tag are visible in the structure. (**B**) Topology diagram for vvUDG. There are a total of 4 β-sheets (β-sheet 1: strands 1 and 2; β-sheet 2: strands 3 and 7; β-sheet 3: strands 4, 6, 8 and 9; β-sheet 4: strands 5 and 10). (**C**) Wiring plot for human UDG (PDBId: 1AKZ). The figure shows the protein sequence overlaid with assigned secondary structure elements for human UDG. (**D**) Topology diagram for human UDG (PDBId: 1AKZ). The figure was prepared using the PDBSUM server [39, 40].

**Figure 2:**
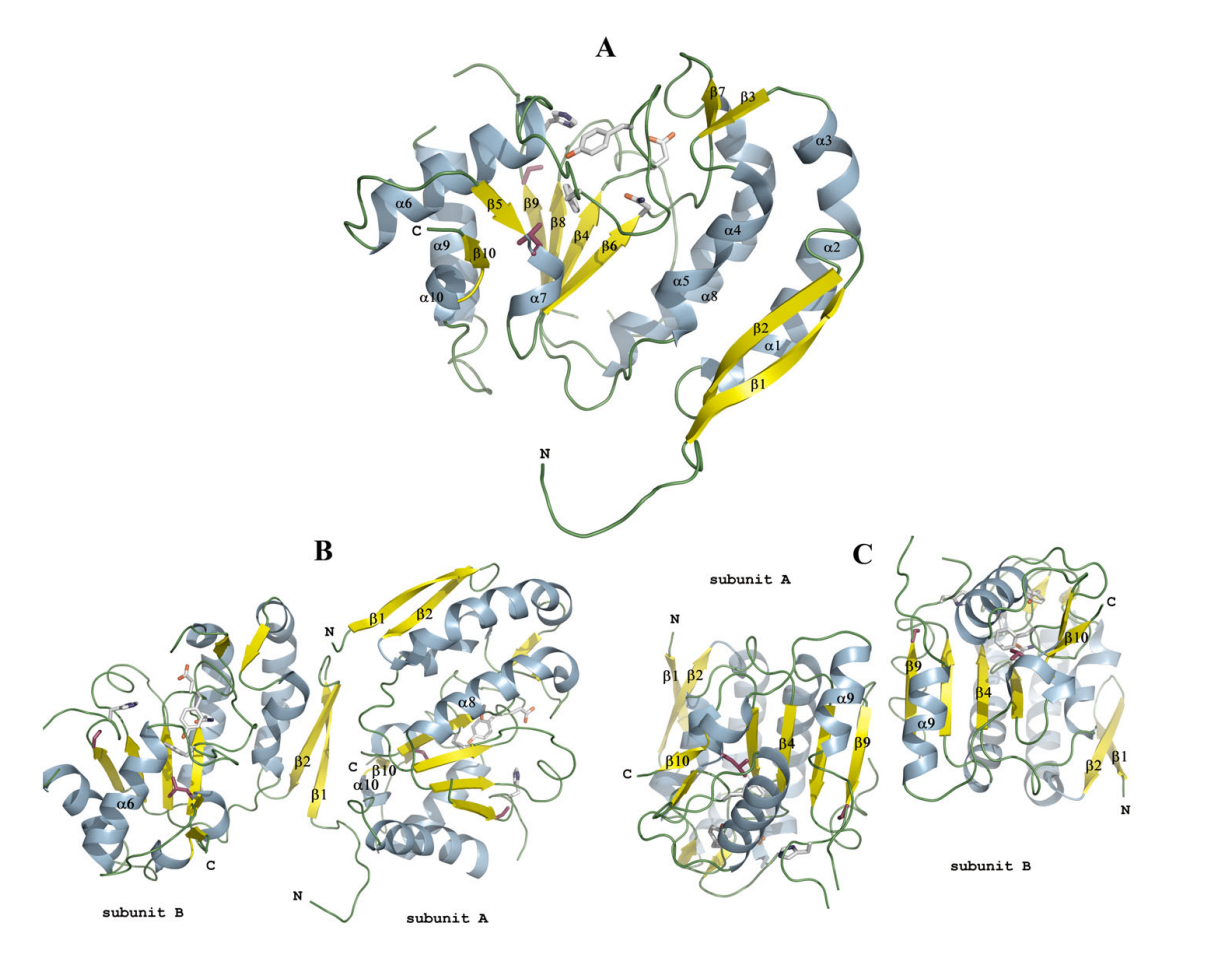
**Ribbon model of vvUDG**. (**A**) Monomer of vvUDG. The figure depicts a ribbon model of vvUDG. The secondary structure elements are labeled according to Fig. 1A. Helices are labeled α1 through α10 and strands are marked β1 through β10. The active site residues are displayed as stick models. Positions of *ts *mutations are also shown as stick models (color code: purple). (**B**) Type I dimer of vvUDG. The figure shows the type I dimer of vvUDG as observed in the asymmetric unit of the trigonal crystal form. The subunits (A and B) in this dimer are related by NCS. Active site residues and *ts *mutation site residues are shown as stick models as seen in (**A**). (**C**) Type II dimer of vvUDG. The figure shows the type II dimer of vvUDG. These dimers are observed in the asymmetric unit of the orthorhombic crystal form (subunits related by NCS) and also in the unit cell of the trigonal crystal form (subunits related by crystallographic symmetry). The active site residues are displayed as stick models. Positions of *ts *mutations are also shown as stick models (color code: purple).

The overall structure of the vvUDG protein in both crystal forms is similar. vvUDG adopts an α/β fold described as DNA glycosylase fold in the SCOP database [6] that is composed of a parallel β-sheet of 4 strands (order 2134) with 3 layers of α/β/α. In Fig. 1 the secondary structure assignments and topology diagrams for vvUDG are compared with those for human UDG. In vvUDG the central β-sheet is surrounded on either side by a total of 5 larger helices. Two helices are observed on one side (residues: 45–49, 50–55; 188–205), and three helices on the other side (residues: 19–32, 33–39; 86–101; 133–149). Additional features are seen at each terminus. At the N-terminus the vvUDG structure exhibits a β-sheet made up of two anti-parallel β-strands (residues: 1–7; 11–17). At the C-terminus the polypeptide chain folds back to form another small anti-parallel β-sheet (residues: 107–109; 215–217) and displays the pairing of two small helices (residues: 110–113; 211–214). The active site groove is visible at the C-terminal edge of the central parallel β-sheet. Active site residues D68, Y70, F79, N120 and H181 are lined up at the edge of the groove. The tertiary structure of a vvUDG subunit is shown in Fig. 2A.

### Assembly and protein-protein interactions

The asymmetric unit in trigonal space group P3_2_21 contains a dimer (type I) with subunits A and B related by non-crystallographic symmetry two-fold symmetry (*rms *deviation of 0.06 Å for all Cα atoms between individual subunits). This NCS related dimer is shown in Fig. 2B. The total buried solvent accessible surface area (SASA) is approximately 4%, corresponding to an interface area of 806 Å ^2 ^between individual subunits. Analysis of protein-protein interactions was performed using the ProFace server [7,8]. In each subunit there are 15 interface residues. For subunit B the contact area is confined to the N-terminal residues 1–4, 6, 9–12 and 14, and residues 38, 45–47 and 54. For subunit A most of the interface residues are found in two regions (residues: 54–56, 58–60; 111–114) with a few additional residues at the termini (residues: 1–2, 16; 211, 214).

The packing of vvUDG in the unit cell of the trigonal form gives rise to a second type of dimer (type II) formed by subunits that are related by crystallographic 2-fold symmetry. The buried surface area in these dimers amounts to 6.5–7% of the total SASA, corresponding to an interface area of 1310 Å ^2 ^between individual subunits. This type II dimer interface has 16–18 residues (167–169; 175–178, 180; 190–191, 194–195, 198, 201–202, 204–206) from each subunit. The contact residues are in the large conserved C-terminal helix 9, the loop (residues 165–167) connecting strands 8 and 9 and in strand 9. Since strand 9 is part of a conserved parallel β-sheet in the central core, interactions involving this strand extend the four-stranded β-sheet to an anti-parallel eight-stranded β-sheet in the dimer. This type II dimer is also observed in the orthorhombic space group P2_1_2_1_2_1 _(Fig. 2C), and is likely the physiological dimer observed in solution. In the orthorhombic crystal form, subunits A through H in the asymmetric unit are arranged as four dimers related by non-crystallographic 2-fold symmetry (*rms *deviation for all Cα atoms between individual subunits is 0.39–0.45 Å). Based on the subunit-subunit interactions these dimers are of type II as seen in the trigonal crystal form.

Considering that vvUDG is a dimer in solution (see Methods) the dimeric assembly in both crystal forms is unlikely to be an artifact of crystal packing. The protein-protein interactions in the dimers may be important in fulfilling vvUDG's role as a component of the DNA polymerase processivity factor. It is tempting to speculate that the interactions in the type I dimer only seen in the trigonal crystal form might mimic the interaction between UDG and A20 in the heterodimeric complex (see later) while the other set of interactions hold the homodimeric assembly of vvUDG.

### Active site

**Figure 3:**
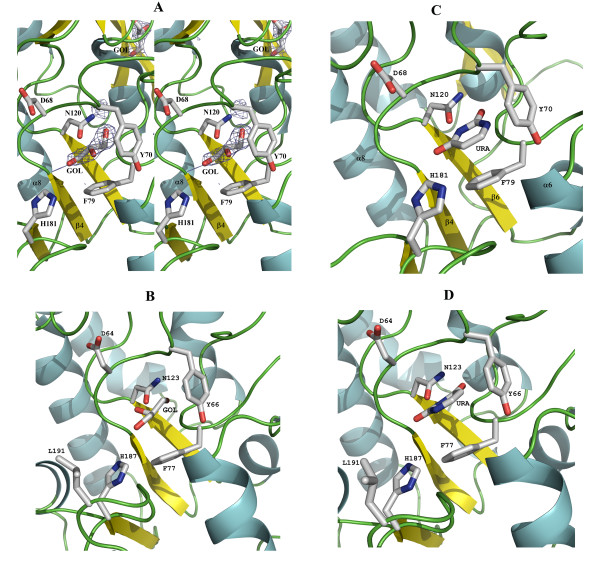
**Superimposition of vvUDG and *E. coli *UDG: Close-up view of the active site**. The figure shows a close-up view of the active site of vvUDG and *E. coli *UDG. The two structures were superimposed with TOPP [22]. (**A**) Active site of vvUDG. The active site of vvUDG with the glycerol (GOL) molecule at the center is shown in this stereo figure. The difference electron density for glycerol (F_o_-F_c _omit map contoured at 3σ) is displayed (blue mesh). A second glycerol molecule away from the active site can also be seen. Active site residues are shown as stick models. (**B**) Active site of *E. coli *UDG (3EUG). Shown is a close-up view of the active site in *E. coli *UDG in the same orientation as the vvUDG in Fig. 3A. The bound glycerol (GOL) in the active site is shown in the center. Active site residues are shown as stick models. (**C**) Active site of vvUDG with modeled uracil. The active site of vvUDG was superimposed on the *E. coli *UDG active site containing uracil. The uracil molecule (URA) is modeled into the active site of vvUDG in the same position and orientation as seen in Fig. 3D for the *E. coli *structure (2EUG). The carbonyl oxygen atoms of uracil in this model superimpose with two hydroxyl groups in glycerol in the vvUDG structure. (**D**) Active site in *E. coli *UDG (2EUG). Shown is a close-up view of the active site in *E. coli *UDG with a bound uracil (URA) molecule in the same orientation as in Figs. 3A and 3B.

vvUDG was crystallized in the absence of any substrate. Glycerol molecules from either the crystallization solution or the cryoprotecting reagent occupied the active site in both crystal forms. The quality of electron density for the active site residues and the glycerol molecules is excellent in each case (see Omit Map; Fig. 3A). Glycerol is an inhibitor of UDG and kinetic studies showed that 200 mM glycerol inhibited the reaction rate of *E. coli *UDG by ~50% [9]. A glycerol molecule (from the cryoprotectant) was located in the uracil binding pocket in the crystal structure of *E. coli *UDG [9]. In this structure the three hydroxyl groups of glycerol mimicked atoms O2, O4, and N3 of uracil (URA) in their interaction with the enzyme. Glycerol forms hydrogen bonds directly or through water molecules with active site residues (Q63, D64, Y66, F77, N123 and H187). In the vvUDG trigonal crystal form, one glycerol molecule is located in the active site of subunit B and occupies the same position as seen in *E. coli *UDG (see Fig. 3B). This glycerol makes interactions with three of the five active site residues (D68, F79 and N120) and their immediate neighbors (see Fig. 3A). In subunit A an imidazole molecule occupies the active site making interactions with D68 and displaying hydrophobic contacts with F79. The chloride ion (Cl^-^) in subunit A shows distances of 3.3–3.5 Å to backbone nitrogen atoms of active site residues Y70 and F79 and the ND2 atom of N120. In subunits A, C, E and G of the orthorhombic crystal form one glycerol molecule is located in each active site and exhibits similar interactions with active site residues D68, Y70, F79 and N120 as described for subunit B of the trigonal crystal form. Details of the contacts involving these ligands in both crystal forms are listed in Table 3.

**Table 3:** Major contacts of ligands with protein residues and water molecules at the active site.

2OWQ:				
**Ligand**	**Atom**	**Residue**	**Atom**	**Distance (Å)**

GOL301 X	O3	D68 B	O	3.37
GOL301 X	O3	Wat146	O	2.83
GOL301 X	O1	N120 B	ND2	3.21
GOL301 X	O1	Wat132	O	2.62
GOL301 X	O1	N120 B	OD1	2.86
GOL301 X	O1	F79 B	N	2.91
Cl201 X	CL	Wat134	O	3.43
Cl201 X	CL	N120 A	ND2	3.26
Cl201 X	CL	Y70 A	N	3.47
Cl201 X	CL	F79 A	N	3.35
IMD401 X	N1	D68 A	O	3.28

2OWR:				

**Ligand**	**Atom**	**Residue**	**Atom**	**Distance (Å)**

GOL605	O1	Y70 A	N	2.99
GOL605	O2	F79 A	N	2.77
GOL605	O2	N120 A	OD1	2.33
GOL605	O2	N120 A	ND2	3.05
GOL612	O1	N120 C	ND2	2.85
GOL612	O3	G76 C	O	2.71
GOL616	O1	Y70 E	N	3.07
GOL616	O1	Wat166	O	3.03
GOL616	O2	N120 E	OD1	2.38
GOL616	O2	N120 E	ND2	2.99
GOL616	O2	F79 E	N	2.65
GOL619	O1	N120 G	OD1	2.69
GOL619	O1	N120G	ND2	2.84
GOL619	O3	D68 G	O	2.62
GOL619	O3	N120 G	OD1	3.01
Cl600	CL	N120 C	N	3.13
Cl600	CL	N120 C	O	2.70
Cl600	CL	GOL612	O2	2.46

Figure 3 shows a comparison of the active sites of vvUDG (with bound glycerol) and *E. coli *UDG complexes (with bound glycerol and uracil). We have modeled a uracil molecule in the active site of vvUDG in an orientation as found in other crystal structures (Fig. 3C).

In both crystal forms additional glycerol molecules are located away from the active site. Contacts formed by non-active site glycerol molecules and other ligands are provided in Additional file 1.

### Variations in active site motifs

**Figure 4:**
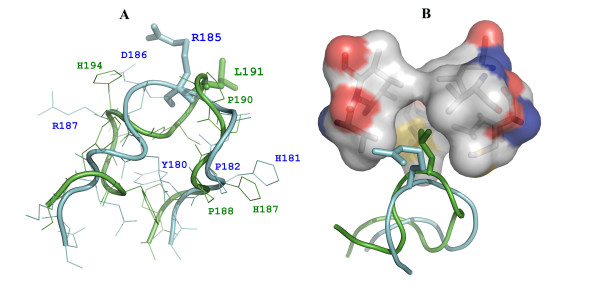
**Comparison of 'Leu intercalation' loops in vvUDG and *E. coli *UDG**. This figure shows a structural comparison of the 'Leu intercalation' loops in vvUDG and *E. coli *UDG and provides a model for the lack of inhibition of vvUDG by Ugi. (**A**) Superimposition of 'Leu intercalation' loops. The two superimposed loops are shown in different colors (vvUDG cyan; *E. coli *UDG green). L191 in *E. coli *UDG (green) and the corresponding residue R185 in vvUDG (cyan) are shown as stick models. Other loop residues are also shown and some of the loop residues are labeled. It can be seen that only the two N-terminal loop residues, Pro (vvUDG P182; *E. coli *UDG P188) and His (vvUDG H181; *E. coli *UDG H187), are identical in sequence and in similar orientations. (**B**) Close-up view of UDG:Ugi complex. The structure of vvUDG was superimposed onto the *E. coli *UDG structure in the UDG:Ugi complex. For the UDG proteins only the loop regions are shown (*E. coli *UDG in green, vvUDG in cyan), while for Ugi the semi-transparent surface of the binding pocket is shown (colored by element). The eight hydrophobic residues of Ugi (M24, V29, V32, I33, V43, M56, L58 and V71) that form the hydrophobic cavity and provide major interactions with the 'Leu intercalation' loop in *E. coli *UDG [19] are shown as stick models. The corresponding residues in the 'Leu intercalation' loop, L191 in *E. coli *UDG (green) and R185 in vvUDG (cyan), are shown as stick models. In *E. coli *UDG:Ugi complex L191 points into the hydrophobic pocket.

The vvUDG sequence exhibits considerable differences in the characteristic motifs utilized by other UDGs for recognizing and flipping the uracil moiety in the substrate DNA during the catalytic activity (see Table 4 for a comparison of motifs in vvUDG with *E. coli *and human UDG). The vvUDG has five of the six conserved active site residues (D68, Y70, F79, N120 and H181), but lacks the conserved Leu residue (see Table 4). In other UDGs the Leu residue is part of the 'Leu intercalation loop', which has the characteristic motif (-HPSPLSXXR-). The 'intercalation loop' (also called catalytic loop) is substantially altered in poxvirus UDGs (see Table 4). Only 3 residues (H181, P182 and R187) match with residues in human and *E. coli *UDG. Residue R185 in vvUDG corresponds to L191 in *E. coli *UDG (see Fig. 4A).

**Table 4:** Characteristic motifs in UDGs (Example: *E. coli *UDG [2EUG] and human UDG [1AKZ]) and variations as seen in vvUDG (2OWQ, 2OWR).

	2EUG	1AKZ	2OWQ, 2OWR
Catalytic water-	62-GQDPYH-67	143-GQDPYH-148	66-GIDPYP-71
activating-loop			
Pro-rich loop	84-AIPPS-88	165-PPPPS-169	-
Uracil specificity	120-LLLN-123	201-LLLN-204	117-IPWN-120
Gly-Ser loop	165-GS-166	246-GS-247	-
Leu intercalation loop	187-HPSPLSAHR-195	268-HPSPLSVYR-276	179-GYHPAARDR-187
Active site residues	D64, Y66, F77, N123, H187, L191	D145, Y147, F158, N204, H268, L272	D68, Y70, F79, N120, H181

Uracil DNA glycosylase catalyzes the hydrolytic cleavage of the N-glycosidic bond of premutagenic uracil residues in DNA by base flipping. Results from a study by Drohat et al. [10] in *E. coli *support a mechanism for catalysis that emphasizes catalytic residue Asp64 as the general base activating a water molecule for nucleophilic attack at C1' of the deoxyribose, and catalytic residue His187 as a neutral electrophile, stabilizing a developing negative charge on uracil atom O2 in the transition state. Stivers et al. [11] demonstrated that base flipping contributes little to the free energy of DNA binding but provides a substantial contribution to specificity through an induced-fit mechanism. For the binding of DNA substrates UDG uses a number of residues that are not part of the active site [12]. According to Tainer et al. [13-15] the DNA repair mechanism of UDG involves pinching of the phosphodiester backbone of damaged DNA using hydroxyl side chains of four conserved serine residues (S88, S166, S189 and S192 in *E. coli *UDG; S169, S247, S270 and S273 in human UDG). This results in flipping of the deoxyuridine from the DNA helix into the enzyme active site. These authors propose that strain induced by serine pinching is used to lower the activation barrier for glycosidic bond cleavage. Results based on S88A, S189A, and S192G "pinching" mutations described by Werner et al. [16] indicated a role for these serine-phosphodiester interactions in uracil flipping and preorganization of the sugar ring into a reactive conformation. The 'Pro-rich' and 'Gly-Ser' loops that contain Ser residues in other UDGs are missing in vvUDG. In addition, the two Ser residues are also missing from the 'Leu intercalation loop' (see Table 4). In the pinch-push-pull uracil detection mechanism, the conserved Leu residue of the 'Leu intercalation loop' penetrates into the DNA minor groove to push the uracil base into the active-site pocket. Based on the structure of the L272A complex of human UDG with DNA, the L272 side chain push is not essential for nucleotide flipping, although it plays a key role in efficient activity [13]. Results of another study [17] suggest that the Leu residue within the -HPSPLS-motif is crucial for the uracil excision activity of UDG.

The side chain of this conserved Leu residue in UDG is also inserted into the hydrophobic cavity of the specific uracil-DNA glycosylase inhibitor (Ugi) from *Bacillus subtilis *[18,19]. Putnam et al. [18] pointed out that a significant fraction of the buried surface area (~10%) in the Ugi-complex results from the complementarity between the conserved Leu residue and the Ugi hydrophobic cavity. Poxvirus UDGs contain instead an Arg residue at this position in the 'Leu intercalation loop' (see Fig. 4A). It was shown that vvUDG activity is not inhibited by Ugi [3]. Superimposition of vvUDG with *E. coli *UDG in the *E. coli *UDG-Ugi inhibitor complex (1UUG) provides a possible explanation for the lack of inhibition (Fig. 4B). Size and electrostatic property of the Arg residue are incompatible with insertion into the hydrophobic cavity of Ugi.

Since the conserved Leu residue and the 'Leu intercalation loop' are critical components for the conventional UDG catalytic mechanism, poxvirus UDGs may utilize a different yet unknown reaction mechanism for carrying out the DNA repair activity.

The conserved motif for uracil specificity (-LLLN-) in UDGs is also altered in the vvUDG protein (-IPWN-). Only N120 as part of the active site residues is conserved. Nonetheless, poxvirus UDG is still highly specific for uracil and does not act on other modified bases [1]. In addition, the 'catalytic water-activating loop' is different in vvUDG. This loop (-GIDPYP-) shows two changes compared to the conserved motif (-GQDPYH-).

### Discussion of the temperature-sensitive Mutants (D*ts*27 and D*ts*30)

**Figure 5:**
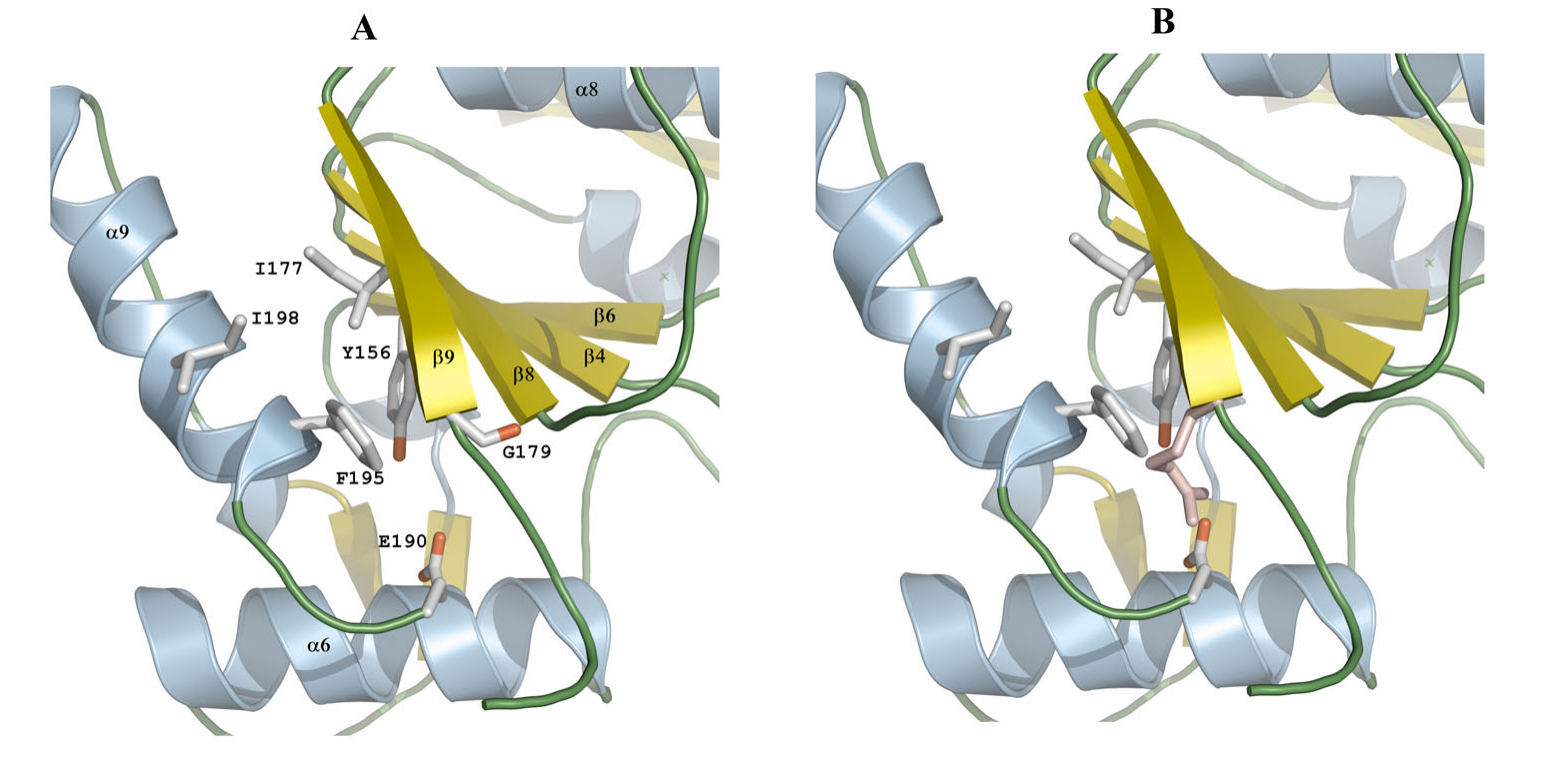
**Model for temperature-sensitive mutant (D*ts*30) of vvUDG**. This figure shows a close-up view of the protein region for the G179R substitution in the temperature-sensitive mutant D*ts*30 and shows the effect of the mutation. (**A**) Residues in this pocket are displayed as stick models and are labeled. (**B**) The pocket is shown in the same view as in Fig. 5A, but an Arg residue (color code: pink) in one rotamer conformation was modeled in place of G179 to indicate that a substitution at this position will introduce steric hindrance. In addition, other rotamer conformations that point towards residues F195 and I198 will position this charged residue even farther into the hydrophobic pocket. In the shown conformation, distances of side chain atoms of the modeled R179 are only 1.5–2.5 Å from surrounding residues.

Two temperature sensitive mutants, which were mapped to the D4 ORF by Ellison et al. [3], have been described. Of these, D*ts*30 (G179R substitution), is of particular interest since this mutation confers defective DNA replication and demonstrates a reduced ability of the mutant D4 protein to interact with A20. Residue G179 is the C-terminal residue of strand 9 in the central parallel β-sheet. As shown in Fig. 5 the substitution of G179 residue by an Arg residue (modeled) would force a large basic residue into the hydrophobic pocket made up of residues Y156, I177, F195 and I198. Y156 is part of the preceding strand 8, I177 is part of strand 9, while F195 and I198 belong to helix 9 following strand 9. Accommodating the side chain of Arg179 will require considerable structural rearrangement. It is likely, although speculative, that the mutation leads to a rearrangement of secondary structure elements (β-strands and α-helices) at and close to the C-terminus to accommodate this residue, which in turn might interfere with binding to A20 and the formation of the A20:UDG processivity factor. Another possibility is a destabilizing effect of this mutation by modulating the dimer interface resulting in a potential interference with the dimer formation.

**Figure 6:**
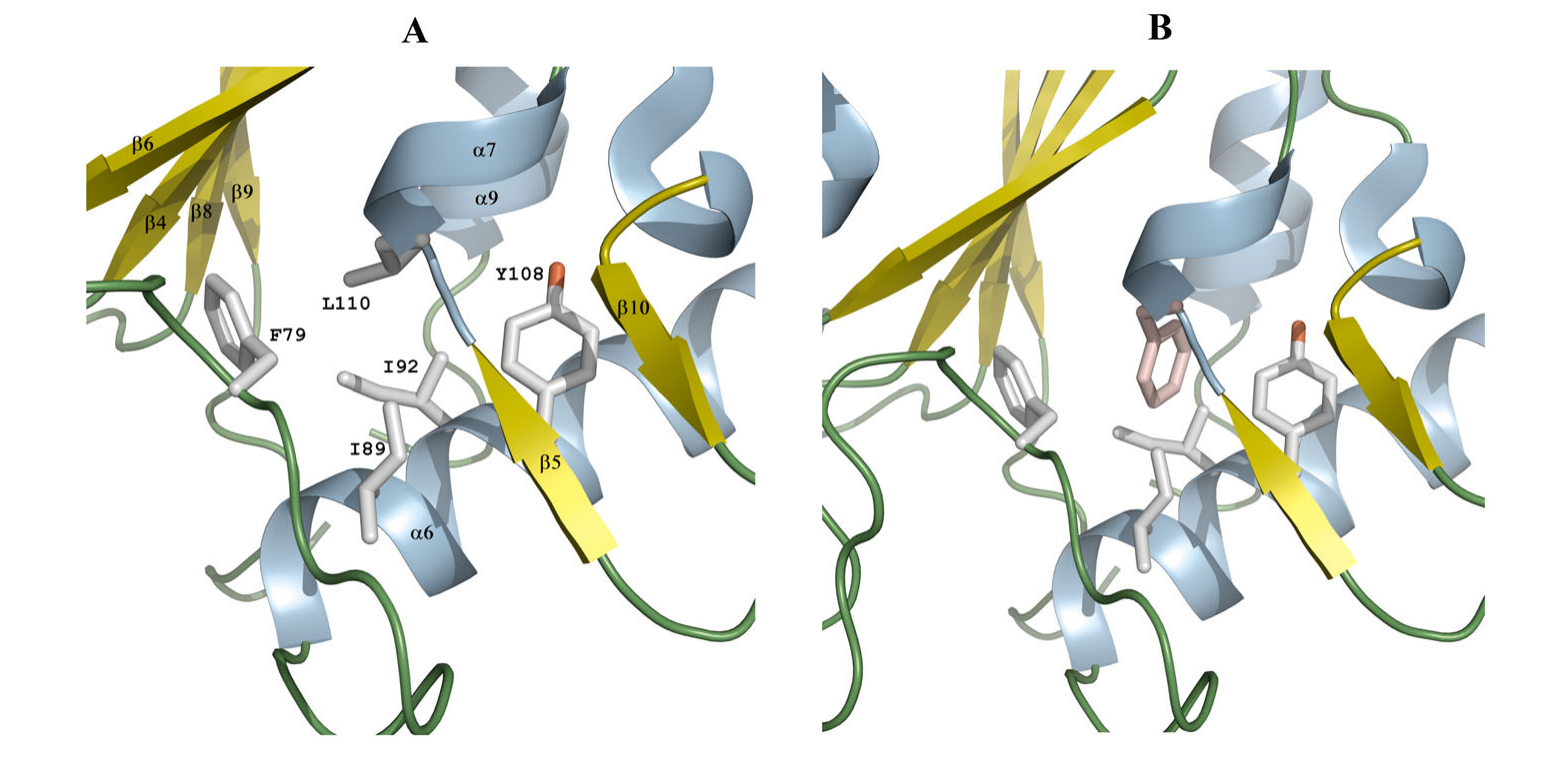
**Model for temperature-sensitive mutant (D*ts*27) of vvUDG**. This figure shows a close-up view of the protein region for the L110F substitution in the temperature-sensitive mutant D*ts27 *and shows the effect of the mutation. (**A**) Residues in this pocket are displayed as stick models and are labeled. (**B**) The pocket is shown in the same view as in Fig. 6A, but a Phe residue (color code: pink) in a preferred rotamer conformation was modeled in place of L110 to indicate that a substitution at this position will introduce steric hindrance. In this conformation, distances of side chain atoms of the modeled F110 are only 2.6–2.8 Å from side chain atoms of I89 and I92.

The other temperature-sensitive mutation, D*ts*27, creates a L110F substitution. Although this residue points into a hydrophobic pocket formed by residues F79, I89, I92 and Y108, the bulky aromatic side chain of the mutated residue is expected to cause steric hindrance and disrupt the local environment as shown in the model in Fig. 6.

### Structural comparison to other UDG structures

vvUDG shows ~20% sequence identity with *E. coli *and human UDGs. Sequence homology with herpes simplex virus1 (HSV1) UDG is also in the same range (21% identity). The structural homology between these proteins is low (*rms *deviation of 2.0 Å for 149 Cα atoms and a match rate of 66% to vvUDG). On the other hand, HSV1 UDG is very similar to human UDG and *E. coli *UDG in terms of sequence (39% identical to human; 49% identical to *E. coli*), fold (*rms *deviation of 1.1 Å for 210 Cα atoms and a match rate of 94% to human; *rms *deviation of 1.2 Å for 199 Cα atoms and a match rate of 88% to *E. coli*) and characteristic motifs [20,21].

The core of the vvUDG structure, however, is similar to other UDGs. The conserved four-stranded central parallel β-sheet, a small second β-sheet made from two anti-parallel β-strands, and six helices in the vvUDG structure match with the observed topology in *E. coli *and human UDG (see Table 5). Superimposition of the vvUDG structure with *E. coli *UDG (PDBId: 2EUG) using program TOPP [22] revealed an *rms *deviation of 2.1 Å for 136 Cα atoms in the matching secondary structure elements (match rate 64.5%; sequence identity 21.3%). The superimposition with human UDG (PDBId: 1AKZ) gave an *rms *deviation of 2.0 Å for 138 Cα atoms in the matching secondary structure elements (match rate 61.9%; sequence identity 20.3%). The vvUDG structure shows some new features that are unique among known UDG structures as discussed earlier and shown in Fig. 1. Compared to vvUDG the C-termini in *E. coli *[9] and human [23] UDG do not show any well defined secondary structure. Other structural differences of vvUDG to *E. coli *and human UDG include fewer α-helices surrounding the central β-sheet, a distinct bend of the N-terminal helix (residues 19–39), some tighter turns in loop regions and the movement of C-terminal residues in strands 8 and 9. These two strands are shifted towards the center of the parallel β-sheet with respect to the other two structures.

**Table 5:** Matching secondary structure elements between *E. coli *UDG (2EUG), human UDG (1AKZ) and vvUDG (2OWQ, 2OWR).

	2EUG	1AKZ	2OWQ, 2OWR
Helix 2, 3	18–31	99–116	19–32, 33–39
Strand 3	36–38	117–119	41–43
Helix 4, 5	40–44, 45–51, 52–56	121–125, 126–130	45–49, 50–55
Strand 4	57–62	138–143	61–66
Helix 6	86–100	167–181	86–101
Helix 7	111–117	192–198	110–113
Strand 6	118–124	199–205	115–121
Strand 7	128–130	209–211	125–127
Helix 8	140–156	221–237	133–149
Strand 8	159–164	240–245	153–159
Strand 9	181–185	262–266	175–179
Helix 9	201–212	282–294	189–205

A structure-based alignment of UDG sequences that included also the vvUDG sequence [13] demonstrates the pitfalls of this approach when the sequence identity drops to about 20%. The new features especially at the termini are not recognized, and the corresponding residues in the viral sequence are instead lined up with the previously observed conserved secondary structure elements in UDGs of other species. It is intuitive that the described novel features in the vaccinia virus UDG structure play a role in the unique function of poxvirus UDG in replication.

**Figure 7:**
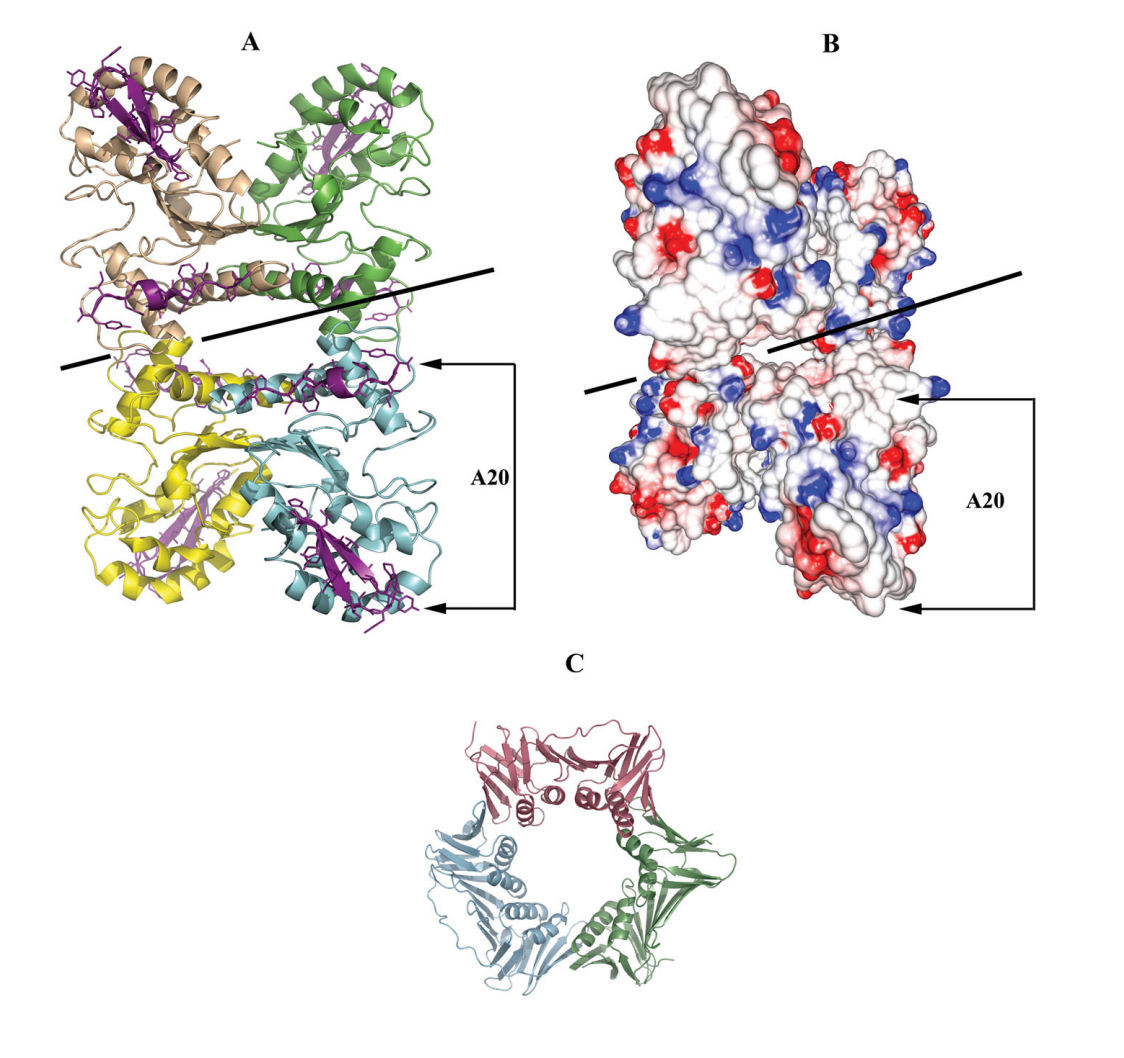
**Model for processivity factor**. This figure shows the potential function of vvUDG as a sliding clamp as part of the viral processivity factor and compares the model with the sliding clamp from *S. solfataricus*. (**A**) A model of the proposed sliding clamp. Shown is a homotetrameric arrangement of two dimers as observed in both crystal structures of vvUDG. The diameter of the central channel is 27 Å. N-terminal residues 1–16 and C-terminal residues 208–218 that are part of the first dimer interface (see Fig. 2B) and implicated in potential binding to A20 are shown and highlighted (purple). The straight line passing through the central channel indicates a DNA molecule. The possible binding site for A20 is shown. (**B**) Model of proposed sliding clamp shown as molecular surface colored by electrostatic potential (color code: red electronegative; blue electropositive; white neutral). The view is the same as in Fig. 7A. The electrostatic potential for the highlighted residues in Fig. 7A indicates neutral regions in these locations. (**C**) Sliding clamp in *S. solfataricus *(2IX2) [25]. The heterotrimeric sliding clamp PCNA in *S. solfataricus *is shown as ribbon model (color code: by chain). The diameter of the central channel is 29 Å.

A plausible model for the function of poxvirus UDG as part of the processivity factor is shown in Fig. 7. The model shows two dimers arranged around a central channel in a homotetrameric arrangement that is observed in both crystal structures (Figs. 7A and 7B). A similar molecular assembly has been noticed in previously studied sliding clamps [24-26]. The diameter of the central channel in vvUDG is approximately 27 Å (distance measured between corresponding residues on either side). This compares well with diameters in the heterotrimeric sliding clamp of PCNA (Fig. 7C) in *Sulfolobus solfataricus *(PDBId: 2IX2) and the homodimeric sliding clamp of the polymerase β-subunit in *E. coli *(PDBId: 1MMI) that are ~29 Å and 30–35 Å, respectively. However, the central channel must have sufficient flexibility in order to accommodate various binding components. In this analogy A20, which acts as a scaffold with binding regions for UDG, E9 and other factors such as D5 and H5 [5], would seem to function as a clamp loader. Protein regions at the type I dimer interface only observed in the trigonal crystal form may be involved in the binding of A20. A part of this dimer interface includes N-terminal residues 1–16 and C-terminal UDG residues 208–218. These 27 residues are almost exclusively hydrophobic. Interestingly, the N-terminal 50 residues of A20 that constitute the minimal interacting binding site for UDG are also predominantly hydrophobic. A hydrophobic interaction in the proposed binding surface is consistent with the observed stability of the heterodimeric A20:UDG complex at high ionic strength (up to 750 mM NaCl) [2]. Although previous experiments have suggested a 1:1 stoichiometry of binding between A20 and UDG [2], the true composition of a functional polymerase unit remains to be determined.

## Conclusion

To our knowledge, vaccinia virus UDG is the only known dimeric protein of this class. We propose that the observed molecular assembly may be related to its cellular functions, which include its role in DNA repair and interaction with one or more binding partners. These interactions are essential to the formation of the processive DNA polymerase needed for the replication of the virus. Discovering tools to disrupt these associations will have tremendous impact in the field of antiviral therapy of poxvirus infection. The structures described here offer a framework for future investigations into the structure of the polymerase complex.

## Methods

### Expression

The D4 gene sequence (AAA48100; Western Reserve 109) encoding for uracil-DNA glycosylase (218 a.a.; Mr ~25 kDa) was subcloned into pET15b vector (Novagen), and transformed into *E. coli *BL21(DE3)pLysS Rosetta cells (Invitrogen). DNA sequencing showed a single substitution (D17N) when compared to the vaccinia virus sequence in the database. The resulting recombinant protein contains a 20-residue insert at the N-terminus comprising of a hexahistidine tag and a thrombin cleavage site. Bacterial cells were grown in Luria-Bertani (LB) broth in the presence of ampicillin to an OD_595 _of 0.7 at 37°C. After induction by isopropyl-β-D-thiogalactopyranoside (IPTG) the protein was expressed for 16 hrs at 18°C. The cell culture was centrifuged at 6400 g for 15 min at 4°C, and cell pellets were stored at -80°C until further use.

### Purification

Frozen cells suspended in a lysis buffer (50 mM Tris, pH 8.0; 100 mM NaCl; 1 mM benzamidine; 0.1 mM phenylmethylsulfonyl fluoride; 5 mM β-mercaptoethanol) were lysed by multiple cycles of freezing and thawing. The cell free extract was prepared by centrifugation at 39200 g for 30 min at 4°C. The recombinant protein was purified from the bacterial extract using affinity chromatography on a Ni-NTA column (Amersham Biosciences). After the protein was eluted from the column with 200 mM imidazole, the hexahistidine tag was removed by treatment with thrombin. The digestion mixture was concentrated and subjected to gel filtration on a Superdex 200 column equilibrated with elution buffer (50 mM Tris, pH 9.0; 100 mM NaCl; 3 mM dithiothreitol). The major portion of the protein eluted as a dimer (calculated from the elution volume of protein standards and from dynamic light scattering experiments). Fractions representing this major peak were concentrated to 8 mg/ml by ultrafiltration.

We also employed a rapid single step affinity purification protocol using a TALON™ (BD Biosciences) column for purification of His-tagged protein from the bacterial extract. Briefly, the cell free extract (pH 7.3) was applied to the TALON™ column containing immobilized cobalt ions. The column was extensively washed with buffer containing 300 mM NaCl and 5 mM imidazole. The tightly bound protein was eluted in a gradient at about 150 mM imidazole in Tris buffer (50 mM, pH 7.3; 100 mM NaCl; 10 mM β-mercaptoethanol). According to the SDS polyacrylamide gel the protein purity was better than 95%. The N-terminal His-tag was not cleaved, and protein in the peak fractions was concentrated to approximately 7 mg/ml without buffer exchange.

### Crystallization and data collection

The thrombin cleaved protein crystallized in two different conditions (condition 1: 5% PEG6000, 7.5% MPD, 0.1 M Hepes, pH 7.25 at 4°C; condition 2: 5% PEG3000, 0.1 M NaCl, 0.1 M Hepes, pH 7.5 at 4°C). Crystals grew to about 0.2–0.3 mm in 1–3 days. These crystals belong to orthorhombic space group P2_1_2_1_2_1 _with unit cell dimensions of a = 117.77 Å, b = 134.06 Å, c = 139.10 Å (see Table 1). In this crystal form, there are 8 subunits of UDG in the asymmetric unit (V_M _~2.7 Å ^3^/Da corresponding to 55% solvent). Results from dynamic light scattering (DLS) verified that the protein exists predominantly as a dimer (estimated MW ~57 kDa, Stokes radius ~3.4 nm).

The protein purified in the one-step procedure without buffer exchange (pH 7.3) was crystallized in 100 mM Hepes buffer, pH 7.25, 12% glycerol and 1.5 M ammonium sulfate as precipitant. The size and quality of crystals were significantly improved using a microseeding protocol (2 μl drops with a 1:1 ratio of protein to seed solution) using the hanging-drop vapor diffusion experiment in NeXtal plates (QIAGEN). The protein crystallized in trigonal space group P3_2_21 with unit cell parameters of a = b = 85.20 Å, c = 139.72 Å, γ = 120° (see Table 1). The asymmetric unit contains two subunits related by non-crystallographic symmetry (V_M _~2.7 Å ^3^/Da corresponding to 54% solvent).

Heavy-atom derivatives were prepared by transferring crystals from seeding experiments into a stabilizing solution (100 mM Hepes buffer at pH 7.25, 12% glycerol and 1.7 M ammonium sulfate) containing in addition varying amounts (1–5 mM) of different heavy atom salts. In this fashion, we obtained successfully a uranyl derivative from an overnight soak in 3 mM uranyl nitrate, which contains U^+4 ^ions in form of the bivalent radical UO_2_^2+ ^group. The dataset of this heavy-atom derivative was isomorphous to the dataset of the native protein with slightly different unit cell parameters (see Table 1). Data were collected at 100 K on cryoprotected (same as crystallization solution but with 25% glycerol) crystals in house (R-Axis IV image plate detector) and at BioCryst (R-Axis IV^++^).

Glycerol was used as cryoprotectant for both crystal forms. For the trigonal form crystals were transferred directly from the crystallization condition (containing 12% glycerol) into the same solution with 25% glycerol, while for the orthorhombic form cryoprotection required a step-transfer protocol (5% to 25% glycerol). All diffraction data were indexed and processed using HKL2000 [27] and DTREK [28] program packages. Diffraction data are summarized in Table 1.

### Structure solution

Efforts to solve the structure by molecular replacement using known UDG structures of *E. coli*, human and herpes simplex virus failed. The structure of vvUDG in trigonal space group P3_2_21 was determined using SHELX [29] by the method of single isomorphous replacement with anomalous scattering (SIRAS) with phase information from a single heavy atom derivative (see Table 1). Isomorphous (to the native dataset) and anomalous differences of the uranyl dataset were good to 2.8 Å resolution. With the help of the graphical interface "hkl2map" determination of the heavy atom substructure (U sites) and initial phasing was successful at 2.8 Å using SHELXD [30]. A correlation coefficient of 46% between E_obs _values (from ΔF) and E_calc _values (from heavy atoms) indicated an excellent quality of this solution. The proper enantiomorph and the right space group (P3_2_21) were clearly established. After density modification and phase extension to 2.5 Å resolution in SHELXE [31] the SIRAS phases for space group P3_2_21 had an overall figure of merit of 0.60 and a connectivity index of 0.91.

The structure of the orthorhombic crystal form was solved by molecular replacement with the program MOLREP [32] using the refined model of the trigonal crystal form. The structure solution shows eight subunits in the asymmetric unit arranged as 4 homodimers.

### Model building and refinement

The obtained reflection file with SIRAS phases was converted to CCP4 mtz format. The resultant map allowed the placement of 59% of the amino acid sequence into the electron density by automated model building using PHENIX [33], and clearly established the presence of the two expected subunits in the asymmetric unit. The remaining residues were fitted into electron density maps calculated with combined SIRAS and model phases. Manual model building was performed with QUANTA (Accelrys, Inc.) and COOT [34]. Model building and stages of subsequent refinement included the use of CNS [35] simulated annealing omit maps. This procedure was especially necessary to follow the chain correctly in the regions of the dimer interfaces because of the close proximity of NCS and symmetry related molecules, and also because these regions contain the residues that are disordered in the final model. In addition, the use of omit maps and difference electron density maps was standard practice throughout model building and refinement. Ligands and water molecules were added using programs CNS [35], REFMAC [36] and COOT [34] and also manually into difference electron density maps (F_o_-F_c _maps, 3σ level). All water molecules that showed low occupancies or high B-factors after refinement and did not satisfy distance constraints for hydrogen-bonding to protein residues were subsequently removed. For the ligands the real space R fit and the quality of electron density was the deciding factor.

Refinement for the trigonal crystal form (subunits A, B) was carried out at 2.4 Å resolution using CNS [35] and REFMAC [36]. Various NCS models (from tight NCS to no NCS) were used during refinement stages. The final NCS restraints produced the lowest R_free _and the smallest difference between R and R_free _values. In addition to restrained refinement by maximum likelihood with tight NCS restraints for main chain atoms (*rms *deviation of distances is 0.18 Å and of B-factors is 0.29 Å ^2^) and medium NCS restraints for side chain atoms (*rms *deviation of distances is 0.50 Å and of B-factors is 0.44 Å ^2^), we also used the TLS refinement option in REFMAC [36]. Each protein subunit (subunits A and B) was divided into three TLS groups (residues 1–97, 98–162 and 163–218) based on analysis by the TLS motion determination (TLSMD) server [37]. Release of NCS restraints and independent refinement of subunits led to an increase in R values and worsened the geometry.

Refinement for the orthorhombic crystal form was carried out at 2.3 Å resolution using CNS [35] and REFMAC [36]. Restrained refinement by maximum likelihood with medium NCS restraints was combined with TLS refinement in REFMAC [36].

Table 2 shows the refinement statistics for both structures. Figures were prepared using PyMOL (DeLano Scientific LLC) and GRASP [38].

Coordinates and structure factors for the trigonal and orthorhombic crystal forms of vvUDG have been deposited in PDB (PDBIds: 2OWQ, 2OWR).

## Authors' contributions

NS was involved in structure determination, refinement and manuscript preparation. AG and AS performed protein expression, purification and crystallization experiments. RK was involved in data collection and data processing. LD and DC were involved in manuscript preparation. DC designed and participated in the experiments, and provided technical and scientific guidance.

All authors read and approved the final manuscript.

## Supplementary Material

Additional file 1 List of major interactions of all ligands in vvUDG. Click here for file
