# Supplementary material for: Crystal structure of vaccinia virus uracil-DNA glycosylase reveals dimeric assembly
Source: BMC Struct Biol. 2007 Jul 2;7:45. doi: 10.1186/1472-6807-7-45 (PMC1936997; doi:10.1186/1472-6807-7-45)
Supplement: Additional file 1 — List of major interactions of all ligands in vvUDG. [file 1472-6807-7-45-S1.pdf]

**Table. List of major interactions of all ligands in vvUDG.**

**2OWQ**

| Ligand | Atom | Residue | Atom | Distance (Å) |
|---|---|---|---|---|
| GOL301 X | O3 | Asp68 B | O | 3.37 |
| GOL301 X | O3 | Wat146 | O | 2.83 |
| GOL301 X | O1 | N120 B | ND2 | 3.21 |
| GOL301 X | O1 | Wat132 | O | 2.62 |
| GOL301 X | O1 | N120 B | OD1 | 2.86 |
| GOL301 X | O1 | F79 B | N | 2.91 |
| GOL302 X | O2 | T12 B | O | 3.41 |
| GOL302 X | O2 | T14 B | N | 3.14 |
| GOL302 X | O2 | T14 B | OG1 | 3.05 |
| GOL302 X | O1 | T14 B | N | 3.33 |
| GOL302 X | O1 | T14 B | O | 2.43 |
| GOL302 X | O1 | F50 B | O | 2.82 |
| GOL303 X | O2 | H181 B | N | 3.47 |
| GOL303 X | O1 | K160 B | N | 2.83 |
| GOL304 X | O1 | Wat111 | O | 3.54 |
| Cl201 X | CL | Wat143 | O | 3.43 |
| Cl201 X | CL | N120 A | ND2 | 3.26 |
| Cl201 X | CL | Y70 A | N | 3.47 |
| Cl201 X | CL | F79 A | N | 3.35 |

**2OWR**

| Ligand | Atom | Residue | Atom | Distance (Å) |
|---|---|---|---|---|
| SO4202 X | O1 | Wat144 | O | 2.66 |
| SO4202 X | O2 | Wat145 | O | 3.22 |
| SO4202 X | O3 | Wat37 | O | 2.83 |
| SO4202 X | O4 | Wat54 | O | 3.06 |
| IMD401 X | N1 | D68 A | O | 3.28 |
| IMD402 X | N3 | H8 B | O | 2.63 |
| IMD403 X | N3 | H8 A | O | 3.39 |
| IMD403 X | N1 | N31 A | O | 3.85 |
| IMD403 X | N1 | Wat123 | O | 3.70 |
| GOL603 | O2 | Y218 A | N | 3.10 |
| GOL603 | O3 | F216 A | O | 3.02 |
| GOL604 | O1 | I135 A | N | 3.04 |
| GOL604 | O1 | S132 A | O | 2.44 |
| GOL604 | O1 | A134 A | N | 2.89 |

| | | | | |
|---|---|---|---|---|
| GOL604 | O2 | D138 C | OD2 | 3.23 |
| GOL604 | O1 | I135 C | O | 2.47 |
| GOL604 | O3 | K131 A | O | 2.54 |
| GOL605 | O1 | Y70 A | N | 2.99 |
| GOL605 | O2 | F79 A | N | 2.77 |
| GOL605 | O2 | N120 A | OD1 | 2.33 |
| GOL605 | O2 | N120 A | ND2 | 3.05 |
| GOL606 | O1 | F49 A | O | 2.68 |
| GOL606 | O1 | Q52 A | N | 3.27 |
| GOL606 | O1 | G76 A | O | 2.58 |
| GOL606 | O2 | G76 A | O | 2.56 |
| GOL606 | O3 | N120 A | O | 2.57 |
| GOL606 | O3 | N120 A | N | 3.04 |
| GOL607 | O1 | E129 A | OE1 | 2.84 |
| GOL607 | O2 | T130 A | O | 2.28 |
| GOL607 | O2 | T130 A | N | 3.11 |
| GOL607 | O3 | Wat488 | O | 2.69 |
| GOL608 | O1 | Y122 A | OH | 2.63 |
| GOL608 | O2 | D162 A | O | 2.53 |
| GOL608 | O3 | K131 A | NZ | 2.89 |
| GOL609 | O3 | S164 A | N | 3.30 |
| GOL609 | O3 | N165 A | N | 2.75 |
| GOL609 | O3 | I166 A | N | 2.73 |
| GOL609 | O3 | D162 A | O | 3.19 |
| GOL610 | O2 | K191 A | O | 2.96 |
| GOL610 | O3 | K191 A | O | 3.31 |
| GOL611 | O1 | N165 C | ND2 | 3.24 |
| GOL611 | O1 | E32 A | OE2 | 3.13 |
| GOL611 | O2 | Wat376 | O | 2.89 |
| GOL611 | O2 | K139 A | N | 3.12 |
| GOL611 | O3 | K139 A | N | 3.27 |
| GOL611 | O3 | Y136 A | O | 2.42 |
| GOL611 | O3 | W137 A | O | 3.14 |
| GOL612 | O1 | N120 C | ND2 | 2.85 |
| GOL612 | O3 | Q52 C | N | 2.89 |
| GOL612 | O3 | F49 C | O | 3.05 |
| GOL612 | O3 | I51 C | N | 3.25 |
| GOL612 | O3 | G76 C | O | 2.71 |
| GOL612 | O3 | T14 C | O | 2.83 |
| GOL612 | O3 | F50 C | O | 3.07 |
| GOL613 | O2 | T14 C | N | 3.14 |
| GOL613 | O3 | T12 C | O | 2.67 |
| GOL613 | O3 | Wat52 | O | 3.30 |

| | | | | |
|---|---|---|---|---|
| GOL614 | O1 | Wat524 | O | 2.89 |
| GOL614 | O2 | E20 C | O | 2.93 |
| GOL614 | O2 | S24 C | N | 3.02 |
| GOL614 | O3 | P21 C | O | 2.71 |
| GOL614 | O3 | Q25 C | NE2 | 2.93 |
| GOL615 | O1 | P46 D | O | 2.96 |
| GOL615 | O1 | F50 D | N | 2.98 |
| GOL615 | O2 | T12 D | O | 2.71 |
| GOL615 | O3 | T14 D | N | 3.00 |
| GOL616 | O1 | Y70 E | N | 3.07 |
| GOL616 | O1 | Wat166 | O | 3.03 |
| GOL616 | O2 | N120 E | OD1 | 2.38 |
| GOL616 | O2 | N120 E | ND2 | 2.99 |
| GOL616 | O2 | F79 E | N | 2.65 |
| GOL617 | O1 | N120 E | N | 3.17 |
| GOL617 | O1 | N120 E | O | 2.86 |
| GOL617 | O2 | G76 E | O | 3.33 |
| GOL617 | O3 | N120 E | ND2 | 2.97 |
| GOL617 | O3 | Wat111 | O | 2.31 |
| GOL618 | O1 | Q146 E | OE1 | 3.26 |
| GOL618 | O2 | L143 E | O | 2.57 |
| GOL618 | O2 | H147 E | N | 3.06 |
| GOL618 | O3 | H147 E | ND1 | 2.70 |
| GOL618 | O3 | K150 E | NZ | 3.25 |
| GOL619 | O1 | N120 G | OD1 | 2.69 |
| GOL619 | O1 | N120 G | ND2 | 2.84 |
| GOL619 | O3 | D68 G | O | 2.62 |
| GOL619 | O3 | N120 G | OD1 | 3.01 |
| GOL620 | O1 | Wat416 | O | 3.04 |
| GOL620 | O1 | G74 G | O | 3.00 |
| GOL620 | O2 | D73 G | N | 3.10 |
| Cl600 | CL | N120 C | N | 3.13 |
| Cl600 | CL | N120 C | O | 2.70 |
| Cl600 | CL | Gol612 | O2 | 2.46 |
| EPE601 | O1S | K160 A | N | 3.18 |
| EPE602 | O2S | A183 E | O | 3.39 |
| EPE602 | O3S | A184 E | N | 3.42 |
